# Aneurysms of pancreaticoduodenal arcade: Clinical profile and endovascular strategies

**DOI:** 10.1002/jgh3.12365

**Published:** 2020-07-08

**Authors:** Supriya Sharma, Raghunanadan Prasad, Archna Gupta, Pranav Dwivedi, Samir Mohindra, Rajanikant R Yadav

**Affiliations:** ^1^ Department of Surgical Gastroenterology Sanjay Gandhi Postgraduate Institute of Medical Sciences Lucknow India; ^2^ Department of Radiology Sanjay Gandhi Postgraduate Institute of Medical Sciences Lucknow India; ^3^ Department of Gastroenterology Sanjay Gandhi Postgraduate Institute of Medical Sciences Lucknow India

**Keywords:** aneurysm, endovascular technique, pancreatoduodenal arcade, rupture

## Abstract

**Background and Aim:**

Pancreaticoduodenal arcade aneurysms (PDAAs) are uncommon lesions associated with a significant risk of rupture and mortality. This study describes the etiology, clinical presentation, and endovascular management strategies of PDAAs across a spectrum of indications.

**Methods:**

The clinical records of patients with PDAAs referred for endovascular management from January 2018 till November 2019 were retrospectively reviewed. Data on presenting symptoms, associated etiologies, and outcomes after endovascular treatment were collected and studied.

**Results:**

We found 15 patients with false and 1 patient with true aneurysm of pancreatoduodenal arcade (PDA). The associated conditions were coeliac artery stenosis, severe necrotizing pancreatitis, and chronic pancreatitis or iatrogenic (postendoscopic papillotomy and percutaneous metallic biliary stenting). The main presenting feature was gastrointestinal bleed, while 2 patients had abdominal pain and 1 had gastric outlet obstruction. A multiphase computed tomography scan demonstrated the ruptured aneurysm in all patients. Site of origin of PDAA influenced the choice of transarterial endovascular strategy (coiling for aneurysms of main trunk of arteries and glue injection for those arising from small arterial branches). This was carried out in an emergency setting for 12 patients and as an elective procedure in 4 patients. Technical success was demonstrated in all patients and clinical success in 14. The two patients who had rebleed were salvaged by repeat endovascular procedure. Postembolization syndrome was seen in three patients.

**Conclusions:**

With advancing technology, endovascular strategies continue to evolve. Careful attention to ensure hemodynamic resuscitation and stability, correction of pre‐existing coagulopathy and attention to technique can lead to the possibility of endovascular approaches as a dependable option in the management of ruptured PDAAs.

## Graphical abstract

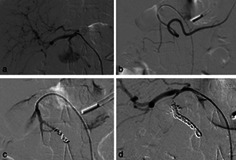

Aneuryms of the pancreatoduodenal arcade are potentially lethal with rising incidence. They can have varied presentations, rupture being potentially life threatening. Endovacsular strategies provide effective minimally invasive management option.

## Introduction

Aneurysms (outpouching) of pancreatoduodenal arcade (PDA) are rare (incidence of 0.1–0.2%).^1^ True pancreatoduodenal arcade aneurysm (PDAA) involves all layers of a thinned but intact arterial wall. They are related to increased retrograde blood flow through the PDA in the presence of a proximal celiac axis obstruction^2^ or acute aortic dissection^3^ or collagen vascular disorders.^4^ False aneurysms of PDA are more common and develop due to disruption of arterial intima and medial layers and do not contain any epithelized wall.^5^ They are seen in acute or chronic infective and/or inflammatory states of pancreas and duodenum, such as severe necrotizing pancreatitis, chronic pancreatitis, and penetrating duodenal ulcers.^6^ Increasing use of endoscopic or percutaneous instrumentation to diagnose and treat hepatopancreatobiliary disease has increased the incidence of iatrogenic pseudoaneurysm.^7^ PDAA have higher rupture rates compared to other visceral artery aneurysms (VAAs).^8^, ^9^ Bergert *et al*. observed that bleeding from the pseudoaneurysms of PDA occurred in 4.6% of their patients with chronic pancreatitis and accounted for 69% of bleeding complications.^6^

Data on the treatment of symptomatic or ruptured PDAA is scarce and can only be extrapolated from available literature on VAAs (any intra‐abdominal aneurysm excluding that of aortoiliac axis). Open surgery has the advantage of effectiveness, durability, reduced need for follow‐up studies, and low mortality rates in elective repairs.^10^ Traditionally, endovascular treatment has been preferred for hemodynamically stable but poor‐risk surgical candidates in the setting of a hostile abdomen when the aneurysm is not easily accessible. The advantages are decreased postoperative pain, wound complications, and length of hospital stay and improved quality of life in the short term.^11^ However, the endovascular approach is fraught with dangers of postembolization syndrome (PES) and incomplete aneurysm exclusion, which causes the patient to remain at risk of rupture.^12^ Kunzle *et al*. report that the endovascular treatment of VAA is associated with a lower incidence of complications and lower median length of hospital stay.^13^ Other studies show equal effectiveness and no difference in operative mortality or decline in renal function between open and endovascular approaches.^14^ Therefore, there is a lack of consensus about the comparative effectiveness of either approach in managing PDAAs.

The current study focuses on the presentation of PDAA, etiology, associated imaging findings, and endovascular strategies in management. We look at technical and clinical success rate of this approach and the need for reintervention.

## Methods

Case records of patients referred for endovascular treatment after being diagnosed/suspected to have PDAA by contrast‐enhanced multiphase abdominal computed tomography (CT) scan from January 2018 to November 2019 were retrospectively reviewed. The diagnosis of PDAA arising from gastroduodenal artery (GDA) and its branches, the gastroepiploic artery, anterior and posterior superior pancreatoduodenal artery, or inferior pancreatoduodenal artery was made by the demonstration of a lesion that was larger in diameter than the size of the native vessel on CT scan. All true and false PDAAs secondary to infective, inflammatory, or percutaneous or endoscopic hepatobiliary intervention were included, excluding postpanceatoduodenectomy GDA stump blowout. The Institutional Ethics Board authorized patient record reviews. Owing to the retrospective nature of the study, patient consent was waived. Information regarding patient demographics, presentation, underlying etiology/comorbidities, imaging findings and classification of the aneurysm as true or false, nature of endovascular procedure, immediate outcomes in terms of cessation of bleeding and need for reintervention, clinical evidence of end‐organ ischemia, per‐procedural complications such as worsening of renal function, and the need for repeat intervention was collated. An upfront surgical approach was not considered in these patients because of our previous experience with endovascular approach. However, our surgery colleagues were always informed of the potential need for rapid exploration and hemostasis in the event of a per‐procedural mishap. The patients were hemodynamically stabilized and coagulopathy corrected.

### *Definitions*

#### *Aneurysm of* *PDA*

Aneurysm (focal dilation of the artery with diameter more than its proximal normal diameter on CECT scan) arising from either the GDA and its right gastroepiploic or superior anterior and posterior pancreaticoduodenal branches and inferior pancreaticoduodenal artery (IPDA).

#### *True* *PDAA* *aneurysm*

Arterial dilatation involves all layers of the wall and absence of radiographically demonstrable infective or inflammatory pathology or a foreign body adjacent to the aneurysm.

#### *False* *PDAA*

False PDAA is the dilatation of any artery of the PDA with demonstration of irregular outline, eccentric location, saccular shape, eccentric thrombosis, and a clear etiology (such as inflammation, collection, presence of stent adjacent to the aneurysm) on imaging.

#### *Technical success*

Technical success was defined as the complete exclusion of aneurysm from systemic circulation after initial intervention.

#### *Clinical success*

Clinical success was defined as relief in symptoms and cessation of overt gastrointestinal bleed (GIB) and no need for reintervention (endovascular or surgical) within 30 days of the primary procedure.

#### *Postembolization syndrome (PES)*

PES was defined as the alteration in biochemical parameters (liver function tests and/or serum amylase) over and above the values before the endovascular intervention.

Procedure‐related complications (minor and major) were graded in accordance with the Society of Interventional Radiology Clinical Practice Guidelines.^15^

### *Radiological intervention*

A multiphase CECT was conducted, and maximum intensity projection and volume‐rendering techniques were used to improve detection of PDAA and site of origin. Anatomical arterial variations and adequacy of collateral flow was assessed to provide a road map for intervention.

Angiographic access was achieved through the right transfemoral route. Using a 0.35″ hydrophilic guidewire (Radio focus; Terumo Inc., Japan) and a Simmons 1 guiding catheter (Cook Medical, Bloomington, Indiana, USA), the celiac artery (CA) and SMA were cannulated under fluoroscopy. Angiograms were obtained to delineate the PDAAs and determine the treatment plan. Angiography of the superior mesenteric artery (SMA) was performed using Cobra or SIM1 catheter (Cook Medical, Bloomington, Indiana, USA) to confirm the presence of a collateral pathway free from aneurysms. A 2.7 Fr Progreat microcatheter (Terumo Inc., Japan) was used to super‐selectively access culprit arteries. Transarterial embolization (TAE) with coils made of platinum (Cook Medical, Bloomington, Indiana, USA) was used for aneurysms arising from the GDA itself. Size (diameter) of the coil was selected to be 20–30% larger than the size of the artery. Coils cause mechanical obstruction, slow blood flow, and induce thrombosis. Due to the rich collateral flow in the PDA, we used the sandwich technique, where occlusion was performed distal, across, and proximal to the neck of the pseudoaneurysm, thus blocking efferent (back door) and afferent arteries (front door). Aneurysm arising from branches of GDA or IPDA were embolized using *N*‐butyl cyanoacrylate (NBCA) glue. This liquid permanent embolic agent polymerizes to form a cast when it comes in contact with anions in blood. Lipiodol® (Geurbet, Villepinte, France), an iodized oil emulsion, was used as a radio‐opaque carrier and diluting agent for glue to slow the rate of solidification. The concentration of glue in the glue–lipiodol mixture determines the rate of polymerization. NBCA: lipiodol was used in a ratio of 1:3. The rate and volume of the injected mixture (inclusive of ≈0.3 mL dead space of microcatheter lumen) was determined by repeated contrast injections to fill the pseudoaneurysm (usually 0.4–0.7 mL) with minimal reflux from the culprit artery. The microcatheter was immediately pulled to prevent attachment to the culprit artery. A new microcatheter was used for further injection if required. Nonionic 5% dextrose was used to flush the catheter before and after glue injection to prevent polymerization within the catheter. A completion angiogram was performed to ensure occlusion of all collateral blood supply to the aneurysm.

## Results

**Figure 1:**
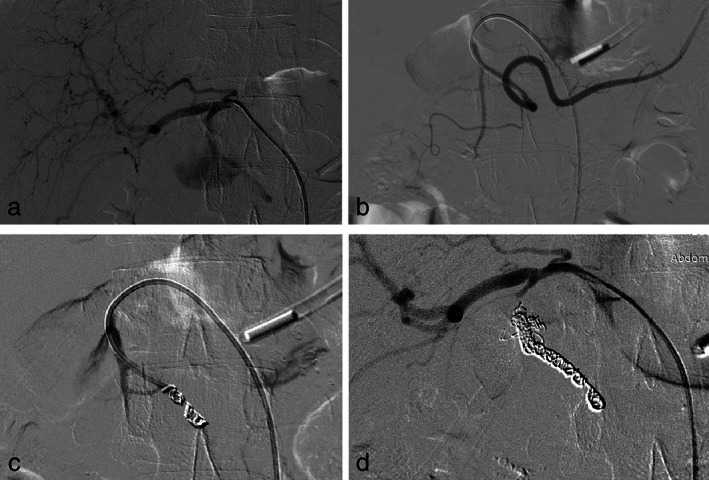
Patient of recurrent acute pancreatitis presented with hemosuccus pancreaticus. Right hepatic artery angiogram taken with a 5Fr Rosch celiac‐1 catheter (a) shows a large pseudoaneurysm arising from the gastroduodenal artery. Angiogram (b) taken after navigating the catheter into the proximal right gastroepiploic artery shows normal right gastro epiploic and superior pancreatoduodenal arteries. Angiogram (c) taken after initial coiling of proximal right gastroepiploic artery shows narrow neck of the pseudoaneurysm. Right hepatic artery angiogram (d) taken after coiling distal and proximal to the pseudoaneurysm neck with 0.035”coils shows no contrast filling in the pseudoaneurysm sac.

**Figure 2:**
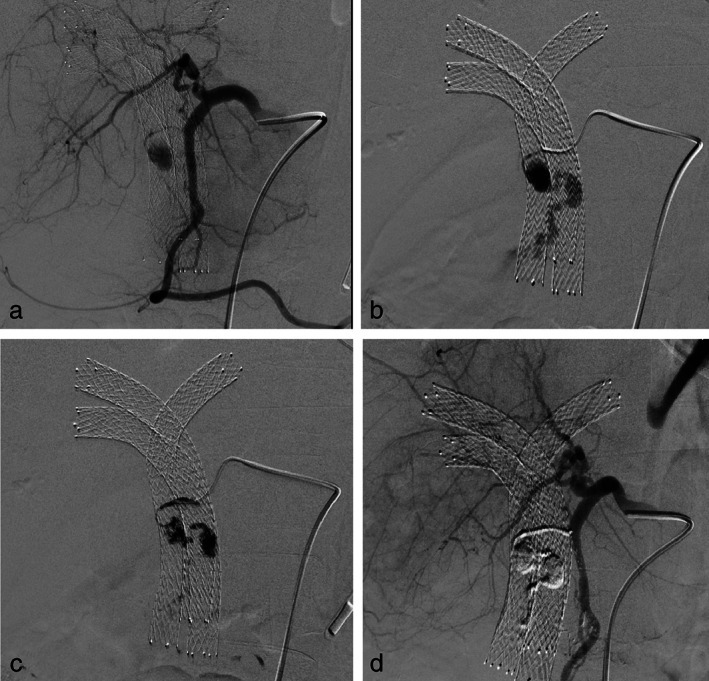
Patient of type IV cholangiocarcinoma palliated with metallic stents in left hepatic duct and right anterior and posterior ducts, presented with melena following rupture of 0.8 × 0.5 cm pseudoaneurysm arising from a branch of the gastroduodenal artery. Common hepatic artery angiogram (a) taken with a Simmons −1, 5Fr catheter shows a pseudoaneurysm arising from a proximal branch of the gastroduodenal artery. Angiogram (b) taken after cannulating the branch artery with a 2.7 Fr Progreat microcatheter shows a pseudoaneurysm with active contrast extravasation. Due to inability to access the artery distal to the pseudoaneurysm with the microcatheter, 0.6 mL of NBCA and lipiodol mixture (1:3 ratio) was injected via the microcatheter (c). Common hepatic artery angiogram (d) taken after NBCA glue injection shows complete obliteration of the culprit artery with exclusion of the aneurysm sac (d). Biliary stents are seen in situ.

There were 16 cases of PDAA (14 males and 2 females) treated by endovascular techniques during the study period. Mean age was 42.8 years (range: 28–70). There was one case of true PDAA associated with celiac artery stenosis, while 15 satisfied criteria for false aneurysms of PDA. Three patients were diagnosed during cross‐sectional imaging for abdominal symptoms or an unexplained drop in hemoglobin; 11 were diagnosed during CECT scan obtained for evaluation of gastrointestinal bleed, while 1 patient each with chronic penetrating ulcer and post‐endoscopic papillotomy (EPT) bleed were detected on digital subtraction angiography (DSA). Six patients had acute necrotizing pancreatitis, three had chronic pancreatitis, and one had a bleed into a pseudocyst. Three patients presented with PDAA bleed following endoscopic control of post‐EPT bleed and one patient had PDAA bleed 2 weeks following percutaneous metallic biliary stenting for hilar cholangiocarcinoma. Etiology of acute pancreatitis was identified as alcohol in three, gallstones in two, and idiopathic in one. There were three cases of rebleed following endoscopic hemostasis of post‐EPT bleeds, and all occurred within 36 h. While gastrointestinal bleed (GIB) in the form of hematemesis or melena was the presenting symptom in 12 patients in our series, two patients had abdominal pain, while one had gastric outlet obstruction (GOO) due to compression of medial wall of the duodenum, and one patient with severe necrotizing pancreatitis with percutaneous drains (PCD) had an unexplained drop in hemoglobin levels. A CECT scan was performed on 14 patients (except 1 patient with suspected penetrating ulcer and 1 patient with post‐EPT bleed) and identified an outpouching in relation to a vessel of the PDAA in 5 patients and hyperdensity in duodenum and pancreatic head or an adjacent collection in 9 patients. Selective angiogram via the CA identified the culprit lesion in 14 cases, while SMA angiogram identified the IPDA pseudoaneurysm in 2 cases. In this series, 12 patients underwent an emergency endovascular procedure, while 4 underwent an elective endovascular procedure. Eight patients underwent transarterial coiling (Fig. 1), seven underwent super‐selective arterial catheterization and glue with lipiodol injection (Fig. 2), and one patient underwent glue injection followed by coil embolization due to rebleed. Demographics, presentation, site, and intervention data have been summarized in Table 1. Technical success with TAE (coil/glue) was seen in all 16 patients and was clinically successful in 14 patients. One patient continued to manifest melena without associated hemoglobin drop due to underlying uremic coagulopathy, and one patient who underwent transarterial glue injection required repeat TAE with coils to arrest the rebleed. Two patients had preprocedural acute kidney injury (AKI), while one patient had chronic kidney disease (CKD). All were maintaining good urine output before the procedure. 1 patient with AKI resolved once the bleeding was controlled, while 1 required a single session of haemodialysis post procedure. The patient with CKD required several sessions of hemodialysis after the procedure. Additional nonendovascular procedures were required in two patients, nasojejunal tube placement in one for GOO, and CBD stenting in one for obstructive jaundice due to persisting compression of lower common bile duct (CBD) because of the collection in the head of the pancreas. None of our patients had clinical or imaging findings to suggest pancreatoduodenal ischemia. No patient developed femoral access site complications. Three patients had transient features of fever and transaminitis, suggesting PES, but recovered spontaneously. None of the patients required a salvage surgical procedure.

**Table 1:** Patient demographics, clinical presentation, location of aneurysm, and type of intervention (*n* = 16)

Age		42.8 years (range 28–70)
Gender (male: female)		14:2
Predominant clinical presentation	Gastrointestinal bleed	13
	Abdominal pain	2
	Gastric outlet obstruction	1
Location of aneurysm in the pancreatoduodenal arcade	Gastroduodenal artery and its branches	14
	Inferior pancreatoduodenal artery and its branches	2
Type of endovascular intervention	Transarterial embolization with coil	8
	Transarterial embolization with glue	8

## Discussion

PDA is an arterial network encircling the pancreatic head and connecting CA and SMA.^16^ There are other collaterals such as the arc of Buhler (an inconstant direct connection between the CA and the SMA), dorsal pancreatic artery, and the arc of Barlow (a collateral pathway within the omentum, between the epiploic arteries of the splenic artery and the SMA).^17^ Circulation through collaterals may prevent organ ischemia after occlusion of either GDA or IPDA but can also lead to new aneurysm formation, necessitating careful radiological follow‐up at 6 months and possibly yearly thereafter.^18^ We had one patient with true PDAA who presented with gastric outlet obstruction, secondary to duodenal compression following rupture of the true aneurysm arising from IPDA. She was treated with glue embolization of the IPDA and nasojejunal tube placement awaiting resolution of the periduodenal hematoma. Most authors currently do not recommend additional treatment with CA stenosis after treating the PDAA.^19^

None of our patients developed pancreatoduodneal ischemia, although PES was seen in three patients, all of whom underwent TAE with glue. There must have been proximal reflux with embolization of small amounts of the glue into the hepatic circulation despite our utmost care with the volume used for injection. All had spontaneous recovery as reported by others.^15^ Patricia *et al*. caution that studies use varied definitions for PES with poorly reported final outcomes.^20^

We had 15 cases of false PDAA. False aneurysms of the PDA related to trauma, infection, or inflammation are more common than true aneurysms.^21^, ^22^ True aneurysms of PDA are increasingly diagnosed due to frequent use of CECT scan for investigating abdominal complaints. Kazuhiro *et al*. have studied hemodynamic simulation of PDAA using an electric circuit model.^23^ They showed that flow volume of the anterior pancreatoduodenal artery and the posterior pancreatoduodenal artery decreases until the CA stenosis progresses up to 50%; thereafter, it increases to up to three times that of the initial volume with retrograde flow. This drastic hemodynamic change in the artery influences the PDA and might cause true PDAA formation.

Any arterial aneurysm can rupture, comparable to a balloon that expands and bursts. VAAs have slow growth rates and do not rupture when small.^20^ However, the nonatherosclerotic, inflammatory, mycotic, or false VAAs have a higher risk of rupture, independent of size.^4^ In pseudoaneurysms, it is structural wall stress, rather than hemodynamic stress, that drives the arterial wall to deteriorate and ultimately rupture.^23^, ^24^ Tulsyan *et al*. describe rupture rates ranging from 2.3 to 18%, with mortality rates ranging from 20 to 100%.^25^ In another series, the mean size at time of rupture was reported to be 9 mm.^26^ Risk factors for rupture of true PDAA are large size or expanding aneurysm and for false PDAA are ongoing inflammatory process or trauma.^8^ Even a small pseudoaneurysm of the PDA constantly exposed to leaking proteolytic enzymes in the setting of pancreatitis or a penetrating duodenal ulcer is more prone to rupture.^22^

Our patients were referred following development of symptoms secondary to ruptured PDAA. PDAAs can rupture into the gastrointestinal (GI) tract, peritoneal cavity, retroperitoneum, biliopancreatic ducts, or pseudocysts.^27^ Twelve of our patients presented with GIB (hematemesis and/or melena), while one had an unexplained fall in hemoglobin due to bleed into a peripancreatic collection. Two patients had abdominal pain, while one developed GOO due to duodenal compression. None of our patients had a free intraperitoneal rupture. Hence, they could be stabilized hemodynamically, and we could consider a nonsurgical approach. Similar to our experience, there are reports of rupture of a submucosal aneurysm resulting in intramural duodenal hematoma and secondary luminal obstruction.^28^ In the review by Yen *et al*. on GDA aneurysms, abdominal pain was the most common presentation, followed by aneurysm bleeding. Hypotension was associated with more than half of the cases presenting with aneurysm bleeding.^29^ In contrast, Habib *et al*. demonstrated that GIB secondary to ruptured GDA aneurysm is the most common clinical presentation, followed by abdominal pain, GOO, hemobilia, and hemosuccus pancreaticus.^30^

Three patients had rebleed following endoscopic control of post‐EPT bleeds. While endoscopic hemostatic measures may achieve immediate control in many cases, these remain susceptible to rebleed, especially in the setting of ongoing inflammatory pathology. Endovascular techniques then offer a minimally invasive approach for effective control of the bleed.

Treatment options for PDAAs are surgical (ligation, primary repair, and total or partial excision with vein patching) or endovascular. PDAA aneurysms are often located behind or within the parenchyma of pancreas and may not be detected during surgery in about 70% of cases. Perrot *et al*. report that three of six patients with PDAA rupture had to undergo emergency pancreatoduodenectomy with its attendant morbidity because visualization was not possible due to the presence of massive retroperitoneal hematomas.^31^ Advantages of endovascular techniques are their minimally invasive nature, shorter recovery time, and lower risk of morbidity and death.^8^ However, TAE may not always be technically feasible because of difficulties in selective cannulation of feeding vessels of the aneurysm and may lack durability due to refilling of the aneurysm from collateral.^32^, ^33^

We used transarterial coiling by sandwich technique for aneurysms arising from larger arteries such as the GDA and IPDA and transarterial glue injection for obliteration of the aneurysms arising from the branches of the GDA or IPDA. All our patients were hemodynamically stable and had normal coagulation parameters before being taken up for TAE. Patricia *et al*. identified 226 aneurysms in the pancreaticoduodenal and gastroduodenal arteries, of which 182 were treated via an endovascular approach and 44 via open surgery. Although comparative inferences were not possible with available data, the systematic review suggests a possible equipoise between open surgery and endovascular repair of VAAs. This may be a true finding or could reflect the rarity of VAAs and the resulting small number of events across studies, making it unlikely to achieve statistical significance. The authors suggest that more complications are likely to occur with the open approach and hence justify a recommendation for pursuing the endovascular approach as a first choice.^20^

Two of our patients had AKI prior to the endovascular procedure, while one patient had pre‐existing CKD. After the procedure, two patients required postprocedure hemodialysis, while one patient had spontaneous improvement. We believe that the successful arrest of the bleed and consequent correction of hemodynamics contributed to preventing further deterioration in patients with AKI and eventual normalization of renal parameters. Only the patient with CKD required more than one session of hemodialysis postprocedure.

Two patients had rebleed following initial arrest of their bleed. One patient had a pseudoaneurysm from the superior pancreatoduodenal artery and was salvaged by repeat TAE with coiling of the GDA. The other patient was a known case of CKD and continued to have intermittent low‐intensity GIB, despite complete occlusion of aneurysm sac, which settled on its own. CKD patients are known to be at a higher bleeding risk secondary to the coexisting uremic platelet dysfunction.^22^

## Conclusion

Refined endovascular technologies have expanded the role of endovascular procedures for the management of PDAAs. Careful attention to technique can lead to the possibility of transarterial embolization technique as a management option for PDAAs with either elective or emergent indications. Longer‐term follow‐up is, however, required to confirm the preference of endovascular procedures as a first‐line therapy for all cases of PDAAs.

## Conflict of interest

The authors declare that there is no conflict of interest.
